# Transitional fossil earwigs - a missing link in Dermaptera evolution

**DOI:** 10.1186/1471-2148-10-344

**Published:** 2010-11-10

**Authors:** Jingxia Zhao, Yunyun Zhao, Chungkun Shih, Dong Ren, Yongjie Wang

**Affiliations:** 1 Key Laboratory of Insect Evolution and Environmental changes, College of Life Sciences, Capital Normal University, Beijing 100048, China

## Abstract

**Background:**

The Dermaptera belongs to a group of winged insects of uncertain relationship within Polyneoptera, which has expanded anal region and adds numerous anal veins in the hind wing. Evolutional history and origin of Dermaptera have been in contention.

**Results:**

In this paper, we report two new fossil earwigs in a new family of Bellodermatidae fam. nov. The fossils were collected from the Jiulongshan Formation (Middle Jurassic) in Inner Mongolia, northeast China. This new family, characterized by an unexpected combination of primitive and derived characters, is bridging the missing link between suborders of Archidermaptera and Eodermaptera. Phylogenetic analyses support the new family to be a new clade at the base of previously defined Eodermaptera and to be a stem group of (Eodermaptera+Neodermaptera).

**Conclusion:**

Evolutional history and origin of Dermaptera have been in contention, with dramatically different viewpoints by contemporary authors. It is suggested that the oldest Dermaptera might possibly be traced back to the Late Triassic-Early Jurassic and they had divided into Archidermaptera and (Eodermaptera+Neodermaptera) in the Middle Jurassic.

## Background

The earwigs (Dermaptera) are familiar insects, often unwelcomed, mainly due to their nocturnal habit, some feeding on decaying matters, emitting foul smell, and an unfounded myth that earwigs would crawl into peoples' ears and penetrate into their brains during sleep. The Dermaptera belongs to a group of winged insects of uncertain relationship within Polyneoptera, which has expanded anal region and adds numerous anal veins in the hind wing [1]. Earwigs are very scarce in the insect fossil record. Nel et al. in 1994 listed only 73 taxa of Dermaptera described, figured or simply mentioned in literature [2]. Even with subsequent addition of 10 species, the fossil record of the Dermaptera stands at 83 species [3,4]. Evolutional history and origin of Dermaptera have been in contention, especially for the fossil earwigs.

**Figure 1:**
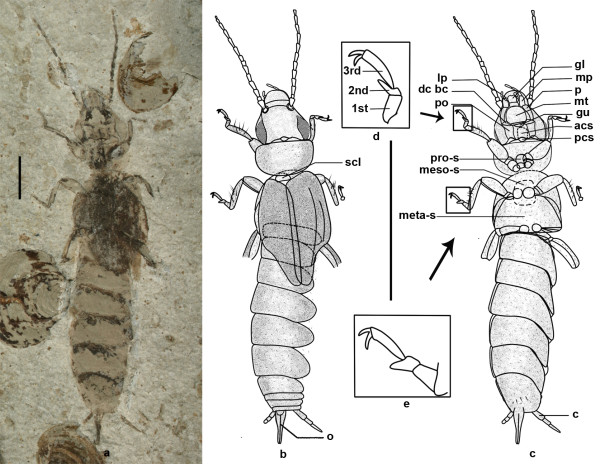
***Belloderma arcuata *gen. et sp. nov., holotype, No. CNU-Der-NN2008002**. a, photo. b, Dorsal view. c, Ventral view. d, Enlarged drawing of foreleg tarsi. e, Enlarged drawing of mid-leg tarsi. Scale bar, 2 mm. scl, scutellum; p, pygidium; o, ovipositor; gl, glossae; mp, maxillary palpus; p, palpifer; mt, mentum; gu, gula; lp, labial palpus; dc and bc, disticardo and basicardo; po, postocciput; c, cercus; acs, anterior cervical sclerites; pcs, posterior cervical sclerites; pro-s, pro-sternum; meso-s, meso-sternum; meta-s, meta-sternum.

**Figure 2:**
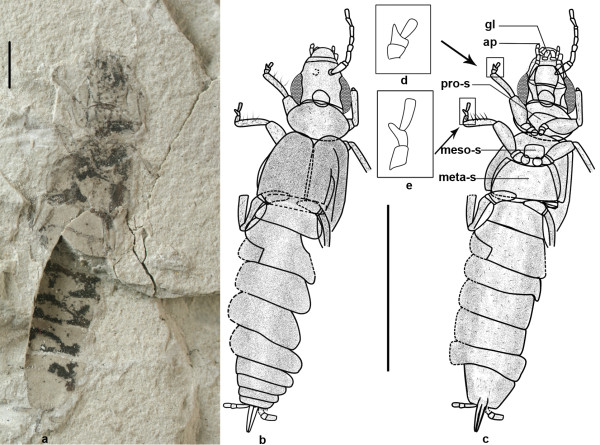
***Belloderma ovata *sp. nov., holotype, No. CNU-Der-NN2008003**. a, photo. b, Dorsal view. c, Ventral view. d, Enlarged drawing of foreleg tarsi. e, Enlarged drawing of mid-leg tarsi. Scale bar, 2 mm. gl, glossa; ap, apical papilla.

Here we report a new genus with two new species (*Belloderma arcuata *gen. et sp. nov. (Figure 1) and *Belloderma ovata *sp. nov. (Figure 2)) in a new family of Bellodermatidae fam. nov., from the Middle Jurassic (Bathonian-Callovian) of the Jiulongshan Formation [5-7] in Daohugou (N41°18'30", E119°13'00") of Ningcheng County, Inner Mongolia, China. Our study of these two earwigs and phylogenetic results provide new understanding of earwigs' origin and evolutional process and enable us to update the phylogenetic and evolutional relationships among major lineages of earwigs.

## Methods

The specimens were examined with a Leica MZ12.5 dissecting microscope and illustrated with the aid of a drawing tube attached to the microscope. Line drawings were made with photoshop9.0 graphics software. Morphological terms used here follow those by Michael S. Engel and Fabian Haas [8].

### Phylogenetic Analysis

The relationships of fossil Dermaptera is re-assessed, the fossil taxa used in the phylogenetic analyses of fossil Dermaptera are listed in Table 1. This study predominantly used body characters, because wing characters and male genitalia characters are almost always poorly preserved in fossils. We followed the original descriptions because these fossils were not available to us. Our phylogenetic analyses of Dermaptera used limited characters to study the reliability and position of the new family Bellodermatidae fam. nov. rather than the inter-relationships of Dermaptera. Thus, if a fossil earwig genus contains several species, only one representative species was chosen to keep the number of taxa low for computational reasons. The character matrix for this dataset is shown in Table 2 and Table 3. We constructed matrices with 17 taxa, 20 characters and 20 taxa, 20 characters separately. The two matrices are the same for the fossil species, but they are different in three additional extant taxa in Table 3. Three species of Blattodea *(Leucophaea maderae, Polyphaga aegyptiaca, Periplaneta americana) *and a species of Plecoptera (*Isoperla obscura*) [9-11] are used as outgroup.

**Table 1:** Extant and fossil taxa used in this study.

Taxon
Blattodea Brunner, 1882
*Leucophaea maderae *(Fabricius, 1775)
*Periplaneta americana *(Linnaeus, 1758)
*Polyphaga aegyptiaca *(Linnaeus, 1758)
Perlodidae
*Isoperla obscura *(Zetterstedt, 1840)
Dermaptera Kirby, 1815
Bellodermatidae fam. nov
*Belloderma arcuata *gen. et sp. nov
Dermapteridae Vishniakova, 1980
*Dermapteron incertaes *(Martynov, 1925)
*Jurassimedeola orientalis *(Zhang, 2002)
*Turanovia incompleta *(Vishnyakova, 1980)
*Sinopalaeodermata neimonggolensis *(Zhang, 2002)
Forficulidae Latreille, 1810
*Forficula auricularia (Linnaeus, 1758)
*Anechura bipunctata (Fabrcius, 1781)
Protodiplatyidae Martynov, 1925
*Archidermapteron martynova *(Vishnyakova, 1980)
*Protodiplatys fortis *(Martynov, 1925)
*Asiodiplatys speciousus *(Vishnyakova, 1980)
*Longicerciata mesozoica *(Zhang, 1994)
*Microdiplatys campodeiformis *(Vishnyakova, 1980)
Pygidicranidae Verhoeff, 1902
*Archaeosoma serratum (Zhang, 1994)
Semenoviolidae Vishniakova, 1980
*Semenoviola obliquotruncata *(Martynov, 1925)
*Semenovioloides capitatus *(Vishnyakova, 1980)
Turanodermatidae Engel, 2003
*Turanoderma sepultum *(Vishnyakova, 1980)

*: only present in matrix of Table 3.

**Table 2:** Character matrix for fossil Dermaptera.

Taxon/character	0	1	2
	1 2 3 4 5 6 7 8 9	0 1 2 3 4 5 6 7 8 9	0
*Leucophaea*	0 0 ? 0 0 0 0 0 0	0 0 0 0 0 0 0 0 0 0	1
*Periplaneta*	0 0 ? 0 0 0 0 0 0	0 0 0 0 0 0 0 0 0 0	1
*Polyphaga*	0 0 ? 0 0 0 0 0 0	0 0 0 0 0 0 0 0 0 0	1
*Isoperla*	1 0 1 1 1 0 0 0 0	0 0 1 0 0 0 0 0 0 0	1
*Archidermapteron*	1 1 1 1 1 0 1 1 1	1 1 0 0 1 0 1 0 0 0	0
*Belloderma *gen. nov	1 0 1 1 1 0 0 1 ?	1 0 1 1 0 1 0 0 0 1	0
*Asiodiplatys*	1 1 0 1 1 0 1 1 1	1 1 0 0 1 0 1 0 0 0	0
*Dermapteron*	1 0 0 1 1 0 0 1 1	1 0 0 0 1 0 0 0 0 0	?
*Jurassimedeola*	1 0 0 1 1 0 0 1 1	1 0 0 0 1 0 0 0 0 0	0
*Longicerciata*	1 1 0 1 1 0 1 1 1	1 1 0 0 1 0 1 0 0 0	?
*Microdiplatys*	1 1 0 1 1 0 1 1 1	1 1 0 0 1 0 1 0 0 0	0
*Protodiplatys*	1 1 0 1 1 0 1 1 1	1 1 0 0 1 0 1 0 0 0	0
*Semenoviola*	1 0 1 1 1 0 0 1 1	1 0 1 0 0 1 0 1 ? 0	?
*Semenovioloides*	1 0 1 1 1 0 0 1 1	1 0 1 0 0 1 0 1 0 0	?
*Sinopalaeodermata*	1 0 0 1 1 0 0 1 1	1 0 0 0 1 0 0 0 0 0	0
*Turanoderma*	1 0 0 1 1 0 1 1 1	1 0 1 0 0 1 0 1 1 0	0
*Turanovia*	1 0 0 1 1 0 1 1 1	1 0 0 0 1 0 0 0 1 0	0

"?": Character not applicable or missing.

**Table 3:** Character matrix for fossil and three extant Dermaptera.

Taxon/character	0	1	2
	1 2 3 4 5 6 7 8 9	0 1 2 3 4 5 6 7 8 9	0
*Leucophaea*	0 0 ? 0 0 0 0 0 0	0 0 0 0 0 0 0 0 0 0	1
*Periplaneta*	0 0 ? 0 0 0 0 0 0	0 0 0 0 0 0 0 0 0 0	1
*Polyphaga*	0 0 ? 0 0 0 0 0 0	0 0 0 0 0 0 0 0 0 0	1
*Isoperla*	1 0 1 1 1 0 0 0 0	0 0 1 0 0 0 0 0 0 0	1
*Archidermapteron*	1 1 1 1 1 0 1 1 1	1 1 0 0 1 0 1 0 0 0	0
*Belloderma *gen. nov	1 0 1 1 1 0 0 1 ?	1 0 1 1 0 1 0 0 0 1	0
*Asiodiplatys*	1 1 0 1 1 0 1 1 1	1 1 0 0 1 0 1 0 0 0	0
*Dermapteron*	1 0 0 1 1 0 0 1 1	1 0 0 0 1 0 0 0 0 0	?
*Jurassimedeola*	1 0 0 1 1 0 0 1 1	1 0 0 0 1 0 0 0 0 0	0
*Longicerciata*	1 1 0 1 1 0 1 1 1	1 1 0 0 1 0 1 0 0 0	?
*Microdiplatys*	1 1 0 1 1 0 1 1 1	1 1 0 0 1 0 1 0 0 0	0
*Protodiplatys*	1 1 0 1 1 0 1 1 1	1 1 0 0 1 0 1 0 0 0	0
*Semenoviola*	1 0 1 1 1 0 0 1 1	1 0 1 0 0 1 0 1 ? 0	?
*Semenovioloides*	1 0 1 1 1 0 0 1 1	1 0 1 0 0 1 0 1 0 0	?
*Sinopalaeodermata*	1 0 0 1 1 0 0 1 1	1 0 0 0 1 0 0 0 0 0	0
*Turanoderma*	1 0 0 1 1 0 1 1 1	1 0 1 0 0 1 0 1 1 0	0
*Turanovia*	1 0 0 1 1 0 1 1 1	1 0 0 0 1 0 0 0 1 0	0
*Archaeosoma	1 0 0 1 1 1 1 1 1	1 0 1 0 0 1 0 1 1 0	?
*Forficula	1 0 1 1 1 1 1 1 1	1 1 1 1 1 2 0 1 1 0	1
*Anechura	1 0 1 1 1 1 1 1 1	1 1 1 1 1 2 0 1 1 0	1

"?": Character not applicable or missing.

All characters were treated as unordered and weighted equally. The data matrices were subjected to the parsimony analyses in NONA and PAUP* (version 4.0b10) [12]. The two programs implement heuristic searches somewhat differently, and so both were employed. Because program NONA can be only defined one outgroup, so we conducted the cladistic analyses by each outgroups respectively. Heuristic search in WinClada/NONA used multiple random additions of taxa and tree-bisection resection branch swapping (options set to hold 10000 trees, perform 1000 replications with one starting tree replication, and the multiple TBR+TBR search strategy). Heuristic search in PAUP* (version 4.0b10) employed a heuristic parsimony analyses, with 1000 random stepwise additions of taxa (TBR branch swapping) under ACCTRAN optimization.

To comply with regulations of the International Code of Zoological Nomenclature (ICZN), we have deposited paper copies of the above article at the Natural History Museum, London; the American Museum of Natural History, New York; the Muséum National d'Histoire Naturelle, Paris; the Russian Academy of Sciences, Moscow; and the Academia Sinica, Taipei.

### List of characters and character states for phylogenetic analysis (*: only present in matrix of Table 3)

1. Head. Opisthognathous (0) or prognathous (1) [13]. The three blattodean species have opisthognathous head, while all other taxa possess a prognathous head.

2. Antennomere. 1st longer than 2nd (0) or 1st shorter than or equal to 2nd (1) (Own observation). Character state 1 is present in *Archidermapteron*, *Asiodiplatys*, *Protodiplatys*, *Microdiplatys*, *Longicerciata*; all other species have character state 0.

3. Hind margin of head. Relatively straight (0) or strongly notched (1) (Own observation). The hind margin of head is strongly notched in *Isoperla*, *Archidermapteron, Belloderma *gen. nov, *Semenoviola*, *Semenovioloides*, *Forficula and *Anechura; the hind margin of head in all other taxa is relatively straight. This character state is uncertain in blattodean species.

4. Neck. Blattoid-type (0) or forficuloid-type (1) [13,14]. The three blattodean species possess a blattoid-type neck; all other examined dermapterans possess a forficuloid-type neck.

5. Shape of pronotum. Disc-like, large, Blattodea-type (0) or disc-like, small, Dermaptera-type (1) [13]. All dermapteran species have disc-like, small, Dermaptera-type pronotum, while being large in three blattodean species.

6. Venation of tegmina. Present (0) or absent (1) (Own observation). Veins are absent in *Archaeosoma, *Forficula and *Anechura; while all other species have veins in their tegmina.

7. Shape of tegmina. Long, outer margin produced into a point (0) or short, apically blunt (1) (Own observation). The tegmina are long and outer margin produced into a point in three blattodean species, *Isoperla*, *Belloderma *gen. nov, *Dermapteron, Jurassimedeola, Sinopalaeodermata, Semenoviola *and *Semenovioloides*; all other have short and apically blunt tegmina.

8. Spiny crest on tegmina. Absent (0) or present (1) [13,14]. The spiny crest is absent in three blattodean species and *Isoperla*, but present in the dermapterans.

9. Hindwing. Long, folded, fan-like (0) or with two transverse folds (1) [13]. A wing package with two transverse folds is present in all fossil and extant Dermaptera, while three Blattodea species and *Isoperla *have a simple, fan-like, folded wing.

10. Broadening. Absent (0) or present (1) [13]. The broadenings are present in all fossil and extant Dermaptera, but is absent in the three blattodean species and *Isoperla*.

11. Spines on femoral carina. Absent (0) or present (1) [13]. Spines are present in *Archidermapteron, Asiodiplatys, Longicerciata, Microdiplatys, Protodiplatys*; they are absent in all other species.

12. Number of tarsomeres. Five (0) or three (1) [13]. There are tarsi with five tarsomeres in three blattodean species, *Archidermapteron, Asiodiplatys, Dermapteron, Jurassimedeola, Longicerciata, Microdiplatys, Protodiplatys*, *Sinopalaeodermata *and *Turanovia*; all other species have 3 tarsomeres.

13. 2nd tarsomeres. Normal, not elongated (0) or heart-shaped (1) [13,14]. All examined species have normal (not elongated) 2nd tarsomeres, except the *Belloderma *gen. nov, *Forficula and *Anechura, which possess a cordiform 2nd tarsomere.

14. Abdominal tergites and sternites. Contiguous (0) or overlapping pleurally (1) [13]. The abdominal tergites and sternites abut in three blattodean species, *Archaeosoma, *Belloderma *gen. nov, *Turanoderma*, *Semenovioloides*, *Semenoviola*; all others overlap pleurally.

15. 8th and 9th abdominal tergites in females. Distinct and separate from 10th tergite (0), narrowed, but separate from 10th tergite and not covered by 7th tergite (1) or fused to 10th tergite and covered by 7th (2) [13]. The tergites are distinct and separate in three blattodean species, *Isoperla, Archidermapteron*, *Asiodiplatys, Dermapteron, Jurassimedeola, Longicerciata, Microdiplatys, Protodiplatys, Sinopalaeodermata *and *Turanovia*; the tergites are narrowed in *Archaeosoma, *Belloderma *gen. nov, *Semenoviola*, *Semenovioloides*, *Turanoderma*; the state 2 is present in *Forficula and *Anechura.

16. Length of cerci. shorter than (0) or longer than abdomen (1) [13]. Character state 1 is present in *Archidermapteron, Asiodiplatys, Protodiplatys*, *Microdiplatys *and *Longicerciata*; character state 0 is present in other species.

17. Adult cerci. Segmented 0) or unsegmented (1) [13]. The adult cerci are unsegmented in *Archaeosoma, Semenoviola, Semenovioloides, *Turanoderma*; *Forficula and *Anechura; all others have segmented cerci.

18. Shape of adult cerci. Straight (0) or evenly curved (1) (Own observation). The cerci are evenly curved in *Turanoderma*, *Turanovia*, *Archaeosoma, *Forficula and *Anechura; the cerci of other species are straight except *Semenoviola*, which is uncertain for this character because of poor preservation.

19. Structure of adult cerci. Symmetrical (0) or asymmetrical (1) (Own observation). The adult cerci are symmetrical in all examined species except *Belloderma *gen. nov, which have asymmetrical cerci.

20. Ovipositor. Exposed (0) or unexposed (1) [13]. The ovipositor is unexposed in outgroup, *Forficula and *Anechura; all other species have an exposed ovipositor, except *Archaeosoma, Dermapteron, Longicerciata, Semenoviola and *Semenovioloides *because the known fossils are male or poor preserved.

## Results

### Description of the specimens

Dermaptera de Geer, 1773

Bellodermatidae Zhao-J, Shih & Ren, fam. nov.

Type genus *Belloderma *Zhao-J, Shih & Ren, gen. nov.

**Diagnosis**: Tegmina elongate along the sutural margin, with costal margin and outer margin strongly arched, while retaining venation. Tarsi three-segmented and forficulid-type. Female with exposed ovipositor. Cerci multi-segmented but short, especially asymmetrical.

***Belloderma ***Zhao-J, Shih & Ren, **gen. nov**.

**Type species: *Belloderma arcuata ***Zhao-J, Shih & Ren, **sp. nov**.

Other species included *Belloderma ovata *Zhao-J, Shih & Ren, sp. nov.

**Diagnosis**: Head relatively large, and posterior margin strongly notched. Antennae with 1st segment broad but shorter than or equal to 3rd. Eyes large. Pronotum transverse, anterior margin almost as wide as posterior one. Mesoscutellum exposed or not. Tarsi shorter than tibiae. Pygidium small. 1st segment of cerci longer than succeeding ones.

**Etymology**: The generic name is a combination of the Latin prefix bello- (meaning pulchritude or beauty) and derma (for Dermaptera). The gender is feminine.

***Belloderma arcuata ***Zhao-J, Shih & Ren, **gen. et sp. nov. (Figure **1)

**Diagnosis**: Pronotum arcuate. Mesoscutellum exposed and semicircular. Pro-sternum quite narrow but elongated longitudinally. Sternum sub-trapeziform.

**Etymology**: The specific name refers to the shape of pronotum.

**Holotype**: CNU-Der-NN2008002 (coll. Shih Chungkun), an almost complete specimen, is housed in the Key Lab of Insect Evolution & Environmental Changes, the College of Life Sciences, Capital Normal University (CNU), Beijing.

**Description**: An adult female, dorsal and ventral aspect. Body (excluding antenna and cerci) 15.5 mm long, covered with pubescence.

Head: 2.3 mm long, 2.6 mm wide, subtriangular. Glossae and paraglossae present. Labial palpi 3-segmented, with apical papilla present on each labial palpus. Maxilla-cardo triangular, with apical palpifer occupying the entire lateral aspect and bearing palpus distally. Right maxillary palpus 4 segments preserved, left one only with first segment preserved. Mentum semicircular with posterior margin straight. Gular behind submentum and nearly semicircular. Laterally, hind portion bearing large subovate eyes, 1.2 mm long, 0.5 mm wide. A pair of protruding tentoria adjacent to eyes. Antennae 13-segmented and 5.1 mm long, 3rd as long as 1st but narrower than 1st, 9th longest. Neck divided into anterior and posterior cervical sclerites, with anterior larger than posterior.

Thorax: Pronotum about 1.8 times as wide as long; anterior margin straight, other margins round. Pro-sternum slightly constricted near coxal cavities of foreleg. Meso-sternum rectangular, with rounded corners. Meta-sternum large and trapeziform, posterior margin wider than anterior one, retrorse.

Legs: Length ratio of foreleg femur: tibia: tarsus is 1.2:1.2:0.9, and 1.3:1.6:1.1 for midleg. Mid-femur nearly as long as or slightly longer than fore-femur, hind-femur longest. Tibiae slender and longer than femora, with bristle on external surface. Tarsi 3-segmented and although not as stout as tibiae, 1st segment long and stout, 2nd shortest, and 3rd longest; pre-tarsal claws well-developed and long, arolium absent. Hind-coxa long, no tibia and tarsus preserved.

Wing: Tegmina about 3 times as long as wide, extending backwards to 2nd abdominal segment; widest aspect lying near basal third, with its outer margin and costal margin strongly arched, a distinct character of the most basal fossil dermapterans, inner margin straight and overlapping. A small distal section of Rs and M visible, other longitudinal veins unclear.

Abdomen: Longer than head and thorax combined. 1st sternum fused to metathorax, with segments 2-7 visible; all segments almost equal in length, with 4th widest. 10 abdominal terga, with 1st and 2nd ones covered by tegmina; 8th and 9th terga distinctly narrow and separated from 10th and not covered by 7th. Asymmetric cerci thin and short, 3-segmented preserved, 3rd acute distally, slightly longer than 1st. Abdomen distally with external ovipositor, 2 mm long, slot present in middle. Pygidium small.

***Belloderma ovata ***Zhao-J, Shih & Ren, **sp. nov**. (**Figure **2)

**Diagnosis**: Pronotum sub-elliptical, anterior margin retrorse and slightly shorter than posterior one. Pro-sternum transverse and almost pentagonal in outline. Sternum transversely rectangular and 7th widest.

**Etymology**: The specific name refers to the shape of the pronotum.

**Holotype**: CNU-Der-NN2008003, housed in the Key Lab of Insect Evolution & Environmental Changes, the College of Life Sciences, CNU, Beijing.

**Description**: An adult female. Body (excluding antenna and cerci) 15.5 mm long, covered with pubescence.

Head: 2.2 mm long, 2.6 mm wide, subtriangular. Paraglossae a pair of cylindrical apical lobes. Labial palpus 3-segmented, with apical papilla present on each labial palpus. Left maxillary palpus 4 segments preserved and 3rd longest, right palpus only with first 2 segments preserved. Mentum and submentum nearly rectangular. Gular sclerite narrow and rectangular. Eyes 1.6 mm long, 0.6 mm wide. Antennae 8-segmented and 3.5 mm long, 3rd slightly shorter and narrower than 1st, length gradually increasing from 4th to 8th. Neck divided into anterior and posterior cervical sclerites.

Thorax: Pronotum about 1.8 times as wide as long. Meso-sternum rectangular, with rounded corners. Meta-sternum large and semicircular. Tegmina poorly preserved and only a small section of M and Cu visible, Cu with two branches (CuA and CuP) with CuP straight and CuA arched.

Legs: Length ratio of foreleg femur: tibia: tarsus is 1.2:1.3:0.5, and 1.4:1.4:0.9 for midleg. Legs almost the same as *Belloderma arcuata *gen. et sp. nov.

Abdomen: Sternum with segments 2-8 visible, 1st segment fused with meta-thorax and absent; segments 3-8 on different faultage with left margin covered; each transversely rectangular and 7th widest. 8th and 9th abdominal tergite are narrower than 2-7 segments. Cerci thin and short, with 3 segments and 1st longest. Ovipositor 1.1 mm long. Pygidium small.

### Remarks

The two species are very similar to each other in body size and eye size, but they differ from each other by shape of the pronotum, pro-sternum and abdominal segments, and they have distinctly different tegmina shapes.

A new family is erected based on these two well-preserved, unique fossil specimens with an unexpected combination of characters (Table 4). The combined characters of this new family allow its allocation to the suborder Eodermaptera: tarsi three-segmented, tegmina retain venation, 8th and 9th abdominal tergite in females are narrowed, but separate from 10th tergite and not covered by 7th tergite and exposed ovipositor. However, there are some particular features of the new family, which are not present for other fossils of Eodermaptera. For instance, the cerci are segmented, which makes the new family be strikingly analogous to the species of suborder Archidermaptera. On the other hand, the new family has a character assemblage similar to extant insects in Neodermaptera, namely, tarsi three-segmented, and 1st segment long and stout, 2nd shortest and distinctly extended distally beneath 3rd one (forficulid-type). The tarsi character is an important family character in the classification of Dermaptera. The species of Dermapteridae of suborder Archidermaptera has 5 tarsi segments for fore, middle and hind legs, i.e. 5-5-5. The tarsi segments of Protodiplatidae of suborder Archidermaptera are 4-4-5, Semenoviolidae and Turanodermatidae of suborder Eodermaptera and all extant earwigs possess tarsi with three tarsomeres, 3-3-3, of which the shape of the 2nd segment is considered as a family character. With respect to tarsi, the new family is close to the modern Dermaptera [1].

**Table 4:** Evolutional trends of key characters for Dermaptera

Characters	Archidermaptera (Protodiplatyidae &Dermapteridae)	Eodermaptera (Bellodermatidae fam. nov.)	Eodermaptera (Semenoviolidae & Turanodermatidae)	Neodermaptera (Extant Earwigs)
No. of hind-leg tarsal segments	5	3	3	3
				
2nd tarsi	Normal, not elongated	heart-shaped	Normal, not elongated	Variable: normal but not elongated; or long and slender; or heart-shaped
				
8th and 9th abdominal tergites in females	Distinct and separate from 10th tergite	Narrowed, but separate from 10th tergite and not covered by 7th tergite	Narrowed, but separate from 10th tergite and not covered by 7th tergite	Fused to 10th tergite and covered by 7th
				
Adult cerci	Segmented	Segmented	Un-segmented forceps. they are segmented in nymphs	Un-segmented forceps. they are segmented in some nymphs
				
Adult cerci	Symmetrical	Asymmetrical	Symmetrical	Symmetrical

No. = Number

In summary, the above-mentioned characters show that the placement of the new family to the suborder Eodermaptera is dubious. To make sure its exact placement, we set up two matrices and carried out phylogenetic analyses, one with only fossil taxa and the other with fossil and three representatives of extant taxa.

### Results of phylogenetic analyses

**Figure 3:**
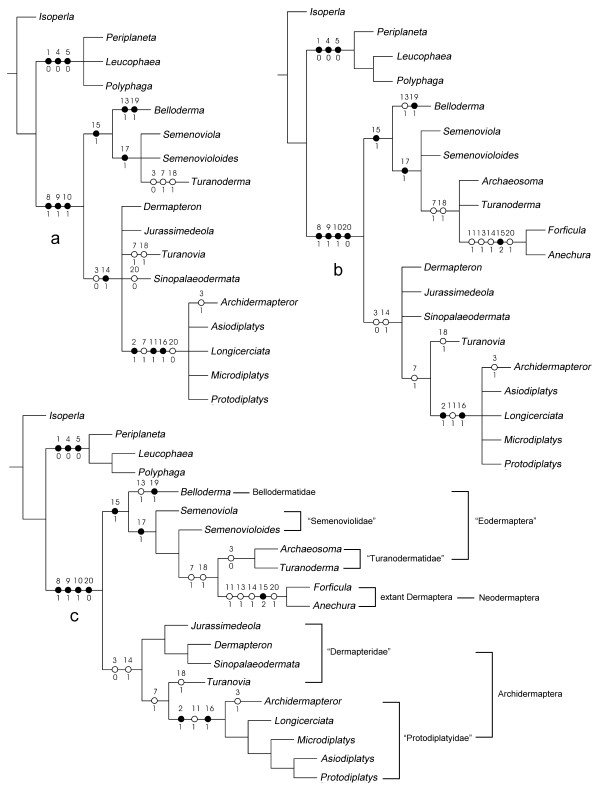
**Phylogenetic analysis (results of NONA with *Isoperla *as outgroup)**. a, Strict consensus tree from Table 2 by NONA (Topology of strict consensus tree by PAUP is same to NONA, see Additional file 1, Fig S1); b, Strict consensus tree from Table 3 by NONA (Topology of strict consensus tree by PAUP is slightly different to NONA, see Additional file 2, Fig S2); c, One most parsimonious tree from Table 3 by NONA. Other results by NONA are same (see Additional file 3, Fig S3).

The phylogenetic analyses of Table 2 by NONA, we get four most parsimonious trees, tree length = 25, consistency index = 0.76, retention index = 0.89 (Figure 3a), and by PAUP, we get 34 most parsimonious trees, tree length = 25, consistency index = 0.7600, retention index = 0.8966 (See Additional file 1). The phylogenetic analyses of Table 3 by NONA, we get two most parsimonious trees, tree length = 31, consistency index = 0.67, retention index = 0.87 (Figure 3b), and by PAUP, we get four most parsimonious trees, tree length = 31, consistency index = 0.6774, retention index = 0.8780 (See Additional file 2). The results by the two programs are similar, and results conducted by NONA with the species of Blattodea as outgroup are same (See Additional file 3). The best supported trees are shown in Figure 3.

Phylogenetic analyses show that the order Dermaptera is divided into two clades: Archidermaptera and Eodermaptera (or Eodermaptera+Neodermaptera, in the analyses including of the extant groups) (Figures 3a, b). The monophyly of Archidermaptera is confirmed in all the analyses, supported by two synapomorphies: straight hind margin of head (Cha. 3: 0) and pleural overlapping of abdominal tergites and sternites (Cha. 14: 1) (Figures 3a, b, c). The suborder Eodermaptera is well assembled in the analyses that is only concerned for the fossil taxa, supported by one synapomorphic character: fusion of 8th and 9th abdominal tergites in females (Cha. 15: 1) (Figure 3a). However in the analysis including the extant representatives, Eodermaptera grouping with Neodermaptera constitute a monophyly, sharing with Cha. 15 (Figure 3c). Although it resulted in some different cladograms, our new family is firmly assigned to the Eodermaptera or (Eodermaptera+Neodermaptera), representing a stem-group of the clade.

## Discussion

### About the suborder Eodermaptera

Earwigs from the Jurassic were usually classified in the extinct suborder Archidermaptera, comprising the known basalmost lineage, which persisted until the earliest Cretaceous [15-18]. The genus *Semenoviola *Martynov, 1925 was placed in Coleoptera when it was erected, then it was transferred to Pygidicranidae Verhoeff, 1902 together with Semenoviolidae and Turanodermidae, which is a family of known Neodermaptera [16]. Besides, as demonstrated by Willmann [17] and Haas and Kukalova-Peck [13], the Semenoviolidae and Turanodermidae are more closely allied to suborder Neodermaptera owing to the unsegmented cerci but excluded from the latter owing to the plesicmorphic retention of venation in the tegmina. For this reason, Engel [19] proposed a new name, Eodermaptera, including Semenoviolidae and Turanodermidae. According to the phylogenetic analyses here, the so-called suborder Eodermaptera becomes paraphyletic, and so we considered that the previously defined Eodermaptera species should be returned to the Neodermaptera or to combine them together as a new classification group.

### About the evolutional history and origin of Dermaptera

Evolutional history and origin of Dermaptera have been in contention, with dramatically different viewpoints by contemporary authors. Some experts suggested the oldest fossils to date are tegmina from the Late Triassic of England and Australia [20], but because of the poor preservation, others suggested the Dermaptera probably originated during early Mesozoic in Asia [21].

Our study of these earwigs and phylogenetic results have shed light on evolutional process and origin. The Dermapteridae species have 5-5-5 tarsi, the same as Protelytroptera, Protodiplatyidae species have 4-4-5 tarsi, and then (Eodermaptera+Neodermaptera) species have 3-3-3 taici. On the other hand, the unique nature of hind wing in Dermapteridae indicates a closer relationship with Protelytridae among Protelytroptera, which is considered as the ancestor of Dermaptera. The afore-mentioned summary shows that Dermapteridae is the most basal in the Dermaptera and they are present in Middle Jurassic. Protodiplatyidae was discovered only from the Late Jurassic [16,18]. The more derived Bellodermatidae fam. nov. was present in the Middle Jurassic of Jiulongshan Formation in Daohugou. Therefore, it is suggested that the oldest Dermaptera might possibly be traced back to the Late Triassic-Early Jurassic and they had divided into Archidermaptera and (Eodermaptera+Neodermaptera) not later than Early Jurassic.

The Turanodermatidae is presently known only from the Late Jurassic of Central Asia but may extend to the Early Cretaceous if the genus *Archaeosoma *[18] proves to be allied [19]. In Figure 3c, phylogenetic analysis shows that character 3 weakly supports the combination of *Archaeosoma *and *Turanoderma *to be Turanodermatidae, but in the strict consensus tree, namely Figure 3b, *Archaeosoma *and *Turanoderma *are paraphyletic, so further research is needed about whether the genus *Archaeosoma *can be allied to the family Turanodermatidae. The previously known eodermapterans up to date have been documented only from the Late Jurassic of Karatau in Chimkent Province of Kazakhstan [13]. The discovery of Bellodermatidae fam. nov. extends the age of previous Eodermapptera to the Middle Jurassic.

**Figure 4:**
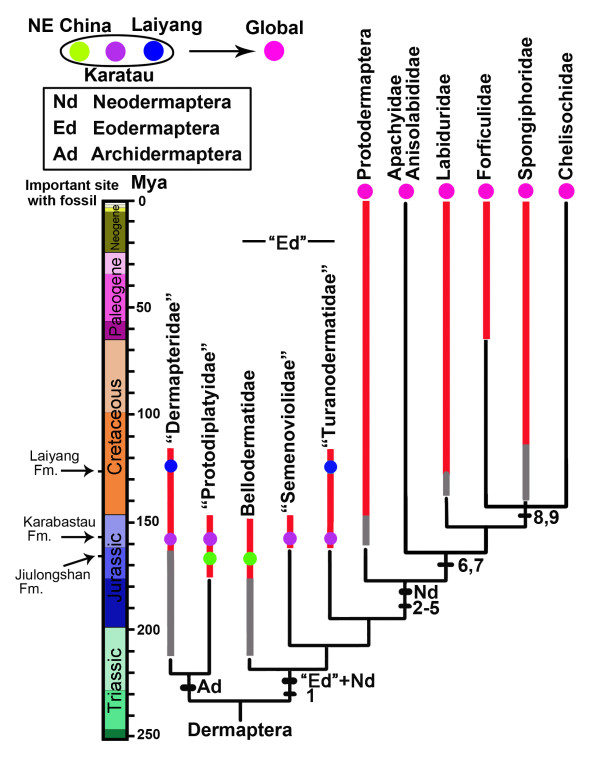
**Phylogenetic relationships among major lineages of earwigs**. Relationships of extant Dermaptera based on Ref. 13. Red lines are the known extinct taxa, blue lines are extant taxa, grayer lines indicate fossils possibly belonging to those groups. Different color dots show different sites with fossils. Significant characters in Dermaptera phylogeny: 1. 8th and 9th abdominal tergites in females narrowed or fused to 10th tergite; 2. Cerci unsegmented, forcipate; 3. Ocelli lost; 4. Tegminal veins lost; 5. Ovipositor reduced; 6. Posterior, ventral cervical sclerite enlarged; 7. Three pygidial subsegments fused; 8. Reduction to single penal lobe and single virga; 9. Expanded regions of anal and intercalary veins distinctly separated.

The previous neodermapterans first appear in the Early Cretaceous [22,23] but might have originated in the Late Jurassic [24]. Certainly, definitive neodermapterans and recognizable pygidicranids are known by the Middle Cretaceous [1,25]. In summary, following Grimaldi and Engel [1], the evolution of Dermaptera is updated in Figure 4.

## Conclusions

This new family Bellodermatidae fam. nov., bridging the missing link between suborders of Archidermaptera and Eodermaptera greatly enhanced the understandings of early evolution of Dermaptera. Based on the phylogenetic analysis, the new family is attributed to the Eodermaptera or (Eodermaptera+Neodermaptera) unambiguously, representing a stem-group of the clade. The suborder Eodermaptera becomes paraphyletic with Neodermaptera, and the previously defined Eodermaptera species should be returned to Neodermaptera or to combine them together. It is suggested that the oldest Dermaptera might possibly be traced back to the Late Triassic-Early Jurassic and they had divided into Archidermaptera and (Eodermaptera+Neodermaptera) in the Middle Jurassic.

## Authors' contributions

JZ carried out the fossil processing, photography, figure preparation, data analysis and interpretation, manuscript drafting and finalization. YZ, CS & DR did the fieldwork, collected the specimens, participated in the data analysis and interpretation, and manuscript modification. YW participated in the data analysis and interpretation, and manuscript modification. All authors read and approved the final manuscript.

## Supplementary Material

Additional file 1 **Fig S1: Strict consensus tree from Table **1** by PAUP**. Result of cladistic analysis by PAUP based on Table 2. Click here for file

Additional file 2 **Fig S2: Strict consensus tree from Table **3** by PAUP**. Result of cladistic analysis by PAUP based on Table 3. Click here for file

Additional file 3 **Fig S3: Analysis with *Periplaneta *as outgroup (Results with *Leucophaea *and *Polyphaga *as outgroup are same)**. a, Strict consensus tree from Table 2 by NONA; b, Strict consensus tree from Table 3 by NONA. Results of cladistic analysis by NONA with *Periplaneta *as outgroup. Click here for file
