# Supplementary figures and images for: Transitional fossil earwigs - a missing link in Dermaptera evolution
Source: BMC Evol Biol. 2010 Nov 10;10:344. doi: 10.1186/1471-2148-10-344 (PMC2993717; doi:10.1186/1471-2148-10-344)

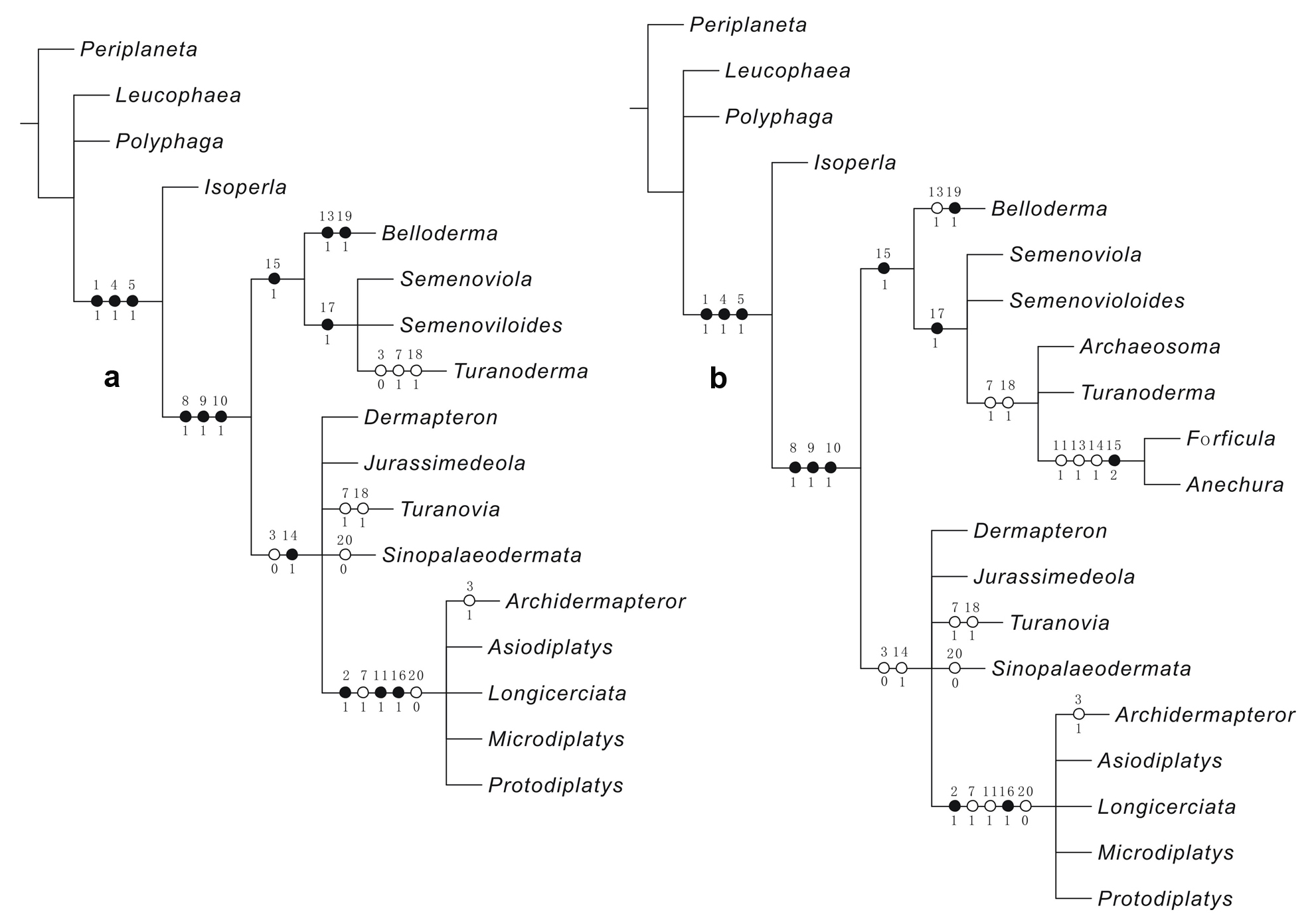

Supplement: Additional file 3 — Fig S3: Analysis with Periplaneta as outgroup (Results with Leucophaea and Polyphaga as outgroup are same). a, Strict consensus tree from Table 2 by NONA; b, Strict consensus tree from Table 3 by NONA. Results of cladistic analysis by NONA with Periplaneta as outgroup. [file 1471-2148-10-344-S3.JPEG]
